# Spatial distribution patterns of ammonia-oxidizing archaea abundance in subtropical forests at early and late successional stages

**DOI:** 10.1038/srep16587

**Published:** 2015-11-13

**Authors:** Jie Chen, Hui Zhang, Wei Liu, Juyu Lian, Wanhui Ye, Weijun Shen

**Affiliations:** 1 Key Laboratory of Vegetation Restoration and Management of Degraded Ecosystems, South China Botanical Garden, Chinese Academy of Sciences, 723 Xinke Rd. Tianhe District, Guangzhou 510650, China; 2 University of Chinese Academy of Sciences, Beijing 100049, China

## Abstract

Characterizing the spatial distribution patterns of soil microorganisms is helpful in understanding the biogeochemical processes they perform, but has been less studied relative to those of macroorganisms. In this study, we investigated and compared the spatially explicit distribution patterns of ammonia-oxidizing archaea (AOA) abundance and the influential factors between an early (ES) and a late successional (LS) subtropical forest stand. The average AOA abundance, vegetational attributes, and soil nutrient contents were mostly greater in the LS than the ES stand (*P* = 0.085 or smaller), but their spatial variations were more pronounced in the ES than the LS stand. The spatial distribution patches of AOA abundance were smaller and more irregular in the ES stand (patch size <50 m) than in the LS stand (patch size about 120 m). Edaphic and vegetational variables contributed more to the spatial variations of AOA abundance for the ES (9.3%) stand than for LS stand, whereas spatial variables (MEMs) were the main contributors (62%) for the LS stand. These results suggest that environmental filtering likely influence the spatial distribution of AOA abundance at early successional stage more than that at late successional stage, while spatial dispersal is dominant at late successional stage.

Soil microorganisms display distinct spatial patterns across environmental patches, at which different biotic and abiotic variables operate with differential contributions^1^,^2^. For instance, the features of distribution patches (e.g., shape and size) and their determinants (e.g., vegetation composition, spatial distance, and soil physicochemical attributes) of soil microbial spatial distribution change with land use types^1^,^3^. Another typical type of environmental patches is resulted from forest succession, and these environmental patches are typically characterized by systematic changes of vegetation type^4^,^5^. However, characterizing the spatial distribution patterns of soil microbes across environmental patches caused by forest succession has received much less attention, even though the importance of spatial analysis approaches has been frequently emphasized in related disciplines such as microbial ecology and soil ecology^1^,^6^. Elucidating the spatial distribution patterns of soil microbial communities in different forest successional stages may help us with the understanding of the recoveries of forest structure and functioning, as soil microbial organisms play a key role in regulating the nutrient biogeochemical cycles that are crucial to ecosystem structure and functioning^7^.

Soil microbes can respond rapidly to shifts in environmental factors, such as soil nutrient content, pH, soil bulk density (BD) and moisture^8^,^9^,^10^. The availability of soil nutrients and the values of soil physical properties have been found to change significantly during forest succession. For instance, soil nitrogen and phosphorus are usually more limited in younger forests during secondary succession due to larger nutrient demand and more frequent disturbances such as timber harvesting and fire^11^,^12^. Soil BD will likely decrease with succession due to the accumulation of soil organic matter (SOM) and the increase of soil water content (SWC)^10^. In addition, the spatial heterogeneity of these environmental factors may also change with forest succession due to the successional changes in vegetation structure such as community composition and canopy cover, which usually cause distinct microenvironment patches under forests^13^,^14^. However, whether the spatial variation of soil microbial communities possesses similar changes with environmental factors during forest succession is largely unknown.

Spatial variables such as distance can limit the dispersal of individuals from one site to another, which therefore influence the assembly patterns of biotic communities^15^. Undoubtedly, distribution of microorganisms can be shaped by dispersal processes, as microbes have a limited habitat range and poor dispersal abilities compared to macro organisms live aboveground^16^. For instance, the similarity of soil microbial community composition decreases with geographic distance^17^. Although the importance of both environmental and spatial variables in shaping microbial communities are evidenced, quantifying the relative contributions of these two group of factors remains a major task to predict the spatial distribution patterns of soil microbes and to link the observed patterns with functional processes^18^,^19^.

Ammonia-oxidizing archaea (AOA) is an important microbial functional group responsible for the oxidation of ammonia (NH_3_) to nitrite (NO_2_^−^)^20^, the first and rate-limiting step in nitrification. AOA and ammonia-oxidizing bacteria (AOB) are the two most important nitrifiers in terrestrial soils, with AOA playing a dominant role over AOB in acid soils on which this study is based^21^,^22^,^23^. Since the main end products of nitrification are nitrate (NO_3_^−^) and nitrous oxide (N_2_O), AOA play important roles in soil fertility, greenhouse gas emission, and nitrogen loss^24^,^25^,^26^. Previous studies have found that AOA abundance and diversity differ among different land use types^1^, vegetation types^27^, farmlands under different management schemes^26^, and environmental gradients such as elevation^28^, precipitation^29^, and salinity^29^. However, only a few studies have quantified the AOA distribution patterns in a spatially explicit fashion^24^,^25^,^26^, which is needed to understand the underlying ecological processes driving the variation of AOA community across spatial scales, and to derive spatially explicit land management strategies for the ecosystem services that soil microbes provide^1^,^26^.

In this study, we analyzed the spatial distribution patterns of the AOA abundance, soil physicochemical properties, and vegetational attributes in an early (ES) and a late successional (LS) subtropical forest stand. These forest stands are typical in southern China and eastern Asia due to long-term anthropogenic disturbances such as deforestation and firewood harvesting. A previous study has found that AOA abundance is significantly positively related to nitrification rates in these forests^22^. Here we focus on the spatial variation of AOA abundance and its determinant factors. Specifically, we aimed to address the following questions: i) Will the magnitude and spatial distribution of AOA abundance differ between the two forest successional stages? ii) How are the spatial variations in AOA abundance related to environmental and spatial factors? iii) What are the relative effects of spatial and environmental factors on the distribution patterns of AOA abundance? We hypothesized that the magnitude and spatial distribution patterns of AOA abundance, and determinant factors would differ between early and late successional forest stands, since many studies have found that soil properties such as microbial biomass and composition, and physicochemical parameters change with forest succession^9^,^30^.

## Results

### General statistics of the AOA abundance and edaphic properties

**Figure 1:**
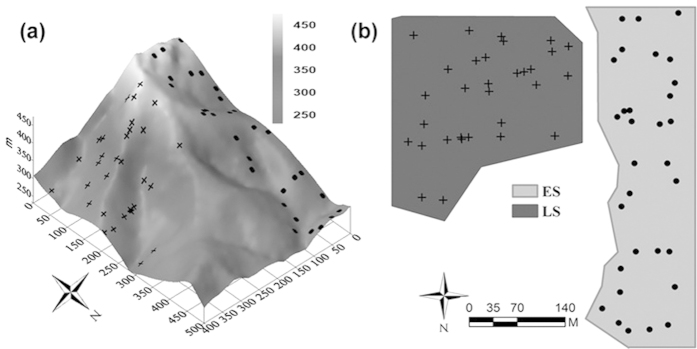
Maps of the two sampling areas divided into early successional (ES) and late successional (LS) stands. (**a**) The 3D map of the 20-ha plot, (**b**) the two-dimensional contour map of our sampling areas. Solid dots and plus signs represent the sampling quadrats in the ES and LS stand.

Averaged over the 31 sampling quadrats (Fig. 1), the AOA abundance in the LS stand was 58% higher than that in the ES stand, although the difference was only marginally significant (*P* = 0.085, Table 1). Similarly, soil chemical constitutes including soil organic matter (SOM), total nitrogen (TN), available nitrogen (AN), total potassium (TK), available potassium (AK) and total phosphorus (TP) were significantly higher in the LS than in the ES stand (*p*  < 0.05, Table 1). However, the values of soil pH, available phosphorus (AP), and bulk density (BD) were greater in the ES stand than in the LS stand (Table 1). Coefficients of variation (cv%) for AOA abundance, BD, TN, AN, AP, and vegetational attributes including Shannon-Wiener diversity, species richness, tree height and diameter at breast height (DBH) were all higher in the ES stand than the LS stand. These results showed that the availabilities of most soil nutrients were higher in the LS stand, and most of the edaphic and vegetational properties displayed higher levels of spatial heterogeneity in the ES stand.

### Spatial distribution patterns of the AOA abundance and environmental variables

**Figure 2:**
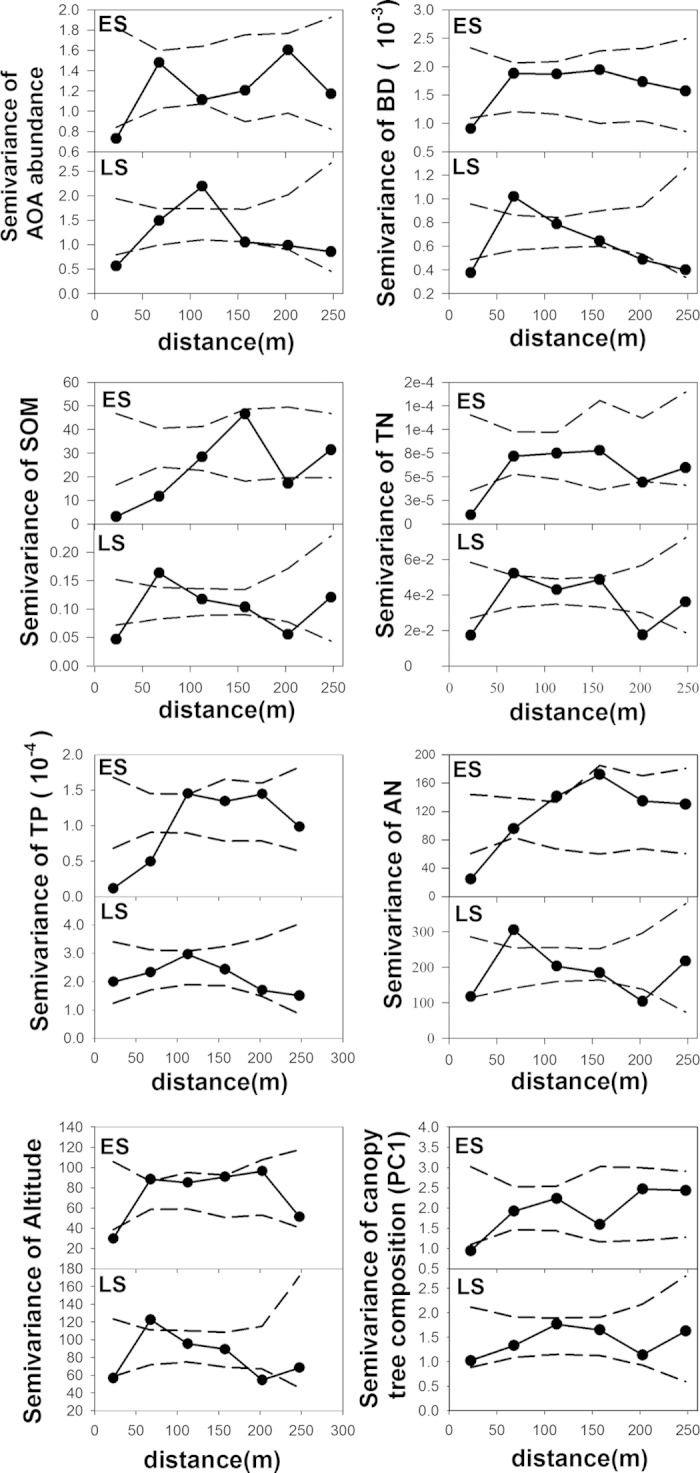
Spatial autocorrelation patterns of AOA abundance (log-transformed archaea *amoA* copy numbers per gram of dry soil) and environmental variables in the ES and LS stands. Observed semivariance is represented by the solid circles, and the dashed lines define the 95% confidence envelope based on 10,000 randomizations of the data. Semivariance values below the confidence envelope indicate positive spatial autocorrelation (less dissimilar than expected at random); values above the confidence envelope indicate negative spatial autocorrelation (more dissimilar than expected at random).

The distribution of AOA abundance was strongly spatially dependent with the normalized sill >78%, (Supplementary Table S1). In the ES stand, the semivariance of AOA abundance showed a positive but saturated relationship with spatial distance, with a significant positive autocorrelation at a separation <50 m (Fig. 2). In the LS stand, the semivariance of AOA abundance displayed a monotonically increasing trend with a nonasymptotic sill, and finally peaked at a separation about 120 m (Fig. 2). Similar to the AOA abundance, most of the edaphic variables showed strong spatial dependence in both stands with the normalized sill >71%, except for AN and TP in the LS stand (Supplementary Table S1). In addition to the positive autocorrelations displayed in the two stands, negative autocorrelations among samples were also observed at separations >100 m in the ES stand for TK and AP, and at separations of 50 to150 m in the LS stand for BD, SOM, TK and AP (Fig. 2, Supplementary Table S1, Fig. S1). Vegetational variables including species diversity, tree density, tree height, DBH and canopy tree composition (veg-PC2) showed no obvious spatial dependence based on the visual inspection of the semivariograms and the failure of the semivariogram model fitting (Supplementary Table S1, Fig. S1); with the exception that canopy tree composition (veg-PC1) showed a positive autocorrelation among samples at a separation <50 m in the ES stand (Supplementary Table S1). The values of altitude and slope were positively autocorrelated in the ES stand, and negative autocorrelation was displayed by altitude in the LS stand, with semivariance peaking at separations between 50 to 100 m (Fig. 2, Supplementary Fig. S1).

**Figure 3:**
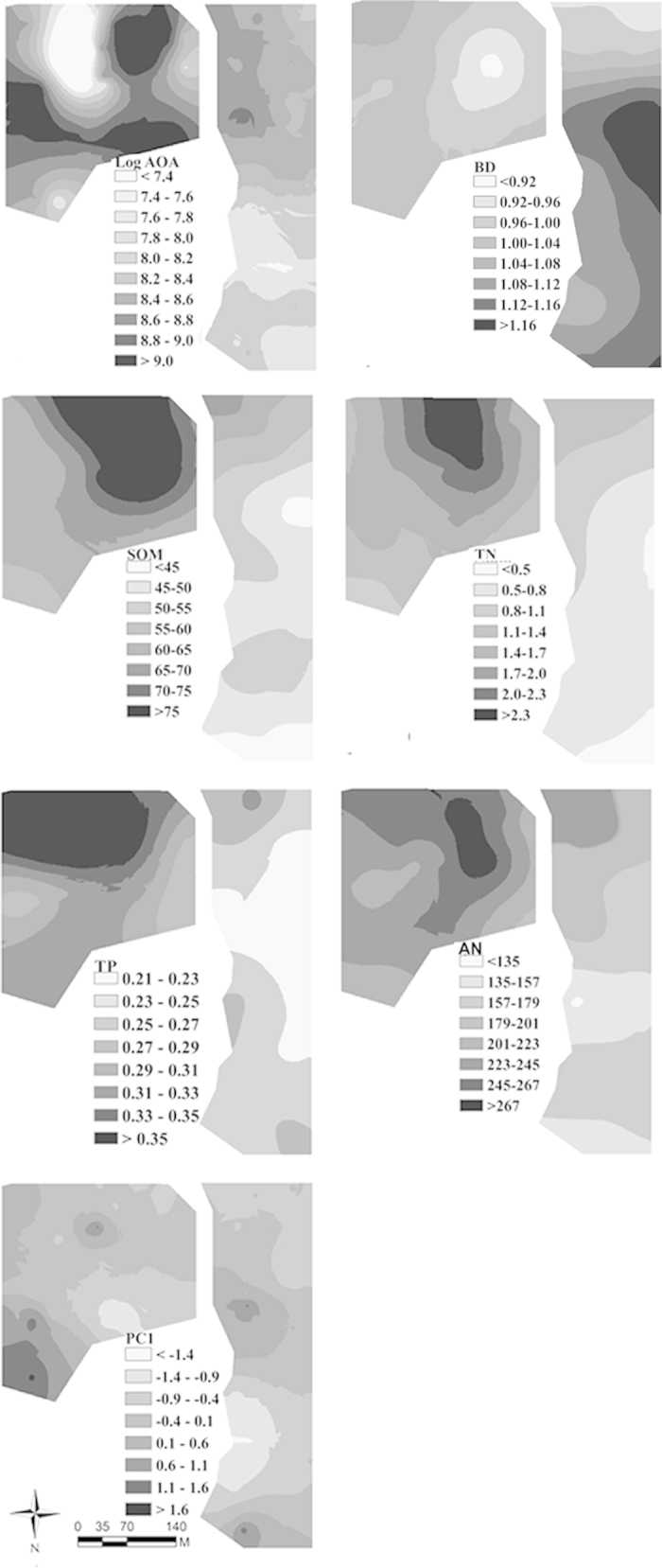
Kriging interpolation maps of AOA abundance (log-transformed archaea *amoA* gene copy numbers per gram of dry soil) and the related environmental variables in the ES and LS stands by using the Geostatistical Analyst Module extension in ArcGIS 10.2 (ESRI Inc., Redlands, CA, USA).

Maps produced by kriging interpolation further illustrated that the distribution patches of AOA abundance were smaller and more irregular in the ES stand than the LS stand (Fig. 3). Overall, AOA was more abundant in the southeast part of the ES stand with higher contents of SOM, TN, TP and AN, and in the southwest and middle areas of the LS stand with lower BD value (Fig. 3). The spatial variation of veg-PC1 displayed nearly the same pattern as that of AOA abundance in the ES stand, while it was patchier and more disorderly than the distribution of AOA abundance and other environmental variables in the LS stand (Fig. 3).

### Relative contributions of environmental and spatial factors to the spatial variations of AOA abundance

**Figure 4:**
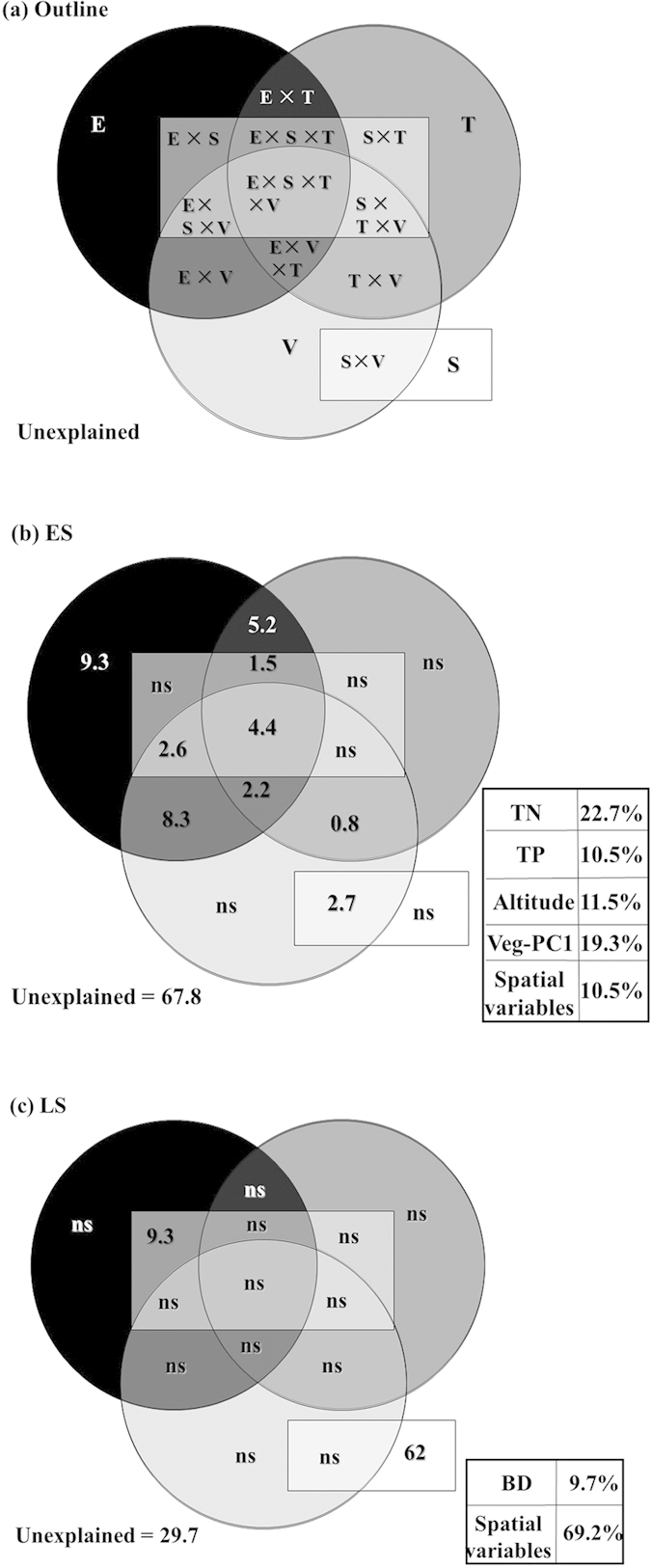
Illustration of the partitioning of the spatial variance in AOA abundance into the relative effects of four groups of environmental factors: edaphic (E), topographical (T), vegetation (V) and the spatial (S), which are included alone or as intersection terms. (**a**) Visualization outline, (**b**) the results of variance partitioning in the ES stand, (**c**) the results of variance partitioning in the LS stand. Significant variables selected in each group are presented in the lower right-hand corner along with the variables contribution to the overall variance, and non-significant parts are indicated as ns.

The spatial, edaphic, topographic and vegetational factors collectively explained 70.3% of the total variation in the distribution of AOA abundance in the LS stand, but only 32.2% in the ES stand (Fig. 4). In the LS stand, the vast majority (62%) of the spatial variation of AOA abundance was attributed to the spatial variables; BD was the only significant environmental variable explaining 9.3% of the AOA variation jointly with spatial variables. In the ES stand, 9.3% of the spatial variation in AOA abundance was explained by the pure effect of edaphic factors, but the pure effects (i.e., non-intersection terms) for the other three groups (i.e., vegetation, spatial, topographical variables) were not detected. Examining the single-factor contribution jointly with other variables revealed that the distribution of AOA abundance in the ES stand was mostly influenced by TN (22.7%) and veg-PC1 (19.3%) and less influenced by altitude (11.5%), TP (10.5%) and spatial variables (10.5%).

## Discussion

The abundance of AOA in the 400-year-old LS stand was 58% higher than in the 60-year-old ES stand. In accordance with the higher AOA abundance, soil organic matter and nutrient contents were also higher in the LS stand (Table 1). These results are in agreement with those of previous studies showing an accumulation of soil carbon, nutrients and the biomass of other microbial communities (e.g., fungi and bacteria) with forest succession/restoration^9^,^30^. Previous researches have also showed shifts in the heterogeneity of environmental factors with forest succession^13^,^14^, although few of them linked the environmental heterogeneity to the successional shifts in soil microbial communities. Here, we found higher values of cv% in AOA abundance in the ES stand (168%) than in the LS stand (108%), suggesting greater spatial variation in AOA abundance in the ES stand. This is also in accordance with most of the edaphic properties (i.e., BD, TN, AN and AP) and vegetational characteristics (i.e., Shannon-Wiener diversity, species richness, tree height and DBH) that had greater cv% in the ES stand, indicating the greater variation of AOA abundance in the ES stand might be related to the higher levels of environmental heterogeneity.

The distribution patches of AOA abundance were smaller in the ES stand, with sample values of AOA abundance being autocorrelated between nearby sampling plots (<50 m). Contrastingly, the AOA abundance distributed along a relatively continuous gradient in the LS stand, with an additional negative autocorrelation peaked at a separation of 120 m. This result supports our hypothesis that the AOA abundance has different spatial distribution patterns between the ES and the LS stands. The successional changes in the composition and structure of important microbial communities during forest restoration/succession have been investigated and proved to be predictable^8^,^9^. However, to our knowledge, this study is the first to examine the spatial distribution patterns of soil AOA community at different forest successional stages. We found that the AOA abundance exhibited nonrandom distribution patterns in both stands. In general, the spatial distribution of AOA abundance in the ES stand was somewhat “spotty” with smaller patches, whereas it was staggered with larger patches in the LS stand (Fig. 3).

The distribution characteristics of AOA abundance in the two stands may be partly attributed to the different environmental conditions defining distinct habitats for soil microbial communities, as environmental factors differ significantly between the ES and the LS stand (Table 1, Table 2). In the ES stand, the AOA distribution was mainly influenced by SOM, TP, TN, AN and veg-PC1, with positive correlations between these factors and AOA abundance being observed (Supplementary Fig. S2) and similar distribution patterns of these factors and AOA abundance being detected (Fig. 2, Fig. 3). Particularly, the quality and quantity of SOM can directly influence its decomposition rate and subsequently the availability of nutrients for AOA communities^31^,^32^. Our results showed that the availability of TP, TN and AN was lower in the early stage of forest succession (Table 1), which might have resulted in the lower AOA abundance in the ES stand. Additionally, soil microbial communities such as AOA may compete with plants for N and P^8^,^11^, leading to a more important role of these two nutrients in shaping the AOA abundance distribution in the ES stand. In contrast to the ES stand, the only important environmental factor affecting the AOA abundance distribution was BD in the LS stand, which had a negative relationship with the AOA abundance (*p* = 0.089, Supplementary Fig. S2). Lower BD in the LS stand means larger soil porosity and more space for storing soil water and oxygen, which are important factors affecting AOA that requires oxygen to oxidize ammonia and water to maintain their activities^33^.

The results of variation partitioning analyses revealed that the relative importance of the spatial and environmental factors in influencing AOA abundance distribution was different between the two stands. The pure effects of environmental factors (i.e., TP, TN, altitude and veg-PC1) could explain 9.3% of the total variations in AOA abundance distribution in the ES stand, whereas no pure effects of spatial factors were detected in this stand. The less soil nutrient availability might have enhanced the sensitivity of the response of AOA community to the changes in these factors within the ES stand. Thus, the environmental patches corresponding to the heterogeneity of these environmental factors can serve as natural filters filtering out the AOA individuals that are not favored by the specific environmental conditions. In this case, the power of dispersal limitation in shaping the AOA community distribution is much weaker compared with that of environmental filtering. On the contrary, the pure effects of spatial factors could explain as much as 62% of the variation in the AOA distribution, and no pure effects of the environmental factors were observed in the LS stand. The higher content of soil nutrients might have provided sufficient substrates and nutrients for the growth of AOA communities in the LS stand; spatial factors such as distance therefore become the main barrier for AOA dispersal from one site to another in this stand^18^. Although BD showed a similar spatial pattern with the AOA abundance in the LS stand, only 9.3% of the variation of AOA abundance distribution could be explained by the spatial arrangement of BD (Fig. 4). The unexplained 29.7% of the variation in AOA distribution in the LS stand may be caused by the spatial arrangement or the pure effects of other environmental variables that we did not measure in this study^34^,^35^. In addition, recent researches have revealed that the differentiation of the relative importance between the environmental and spatial components within a variation partitioning analysis may not be as straightforward as previously thought^36^. The pure effects of spatial factors may be overestimated due to the lack of important spatially structured environmental predictors^36^, which might be the case as what we found in the LS stand. However, the semivariograms and kriging maps in this study showed strongly spatial dependence of AOA abundance in the LS stand, and the spatial pattern of AOA abundance could not be related to most of the environmental variables measured in this study. This may further support our conclusion that the central mechanism determining the distribution of AOA abundance in the LS stand is most likely to be the dispersion restriction caused by spatial variables.

## Conclusions

This study found that the abundance and the spatial distribution pattern of AOA communities differed between the early and late successional EBLF stands. The AOA communities were more abundant and less spatially structured in the late than in the early successional stand. Most of the spatial variations in AOA abundance could be explained by the variations of environmental conditions such as TN, TP, canopy tree composition and their intersections in the early successional stand, but by spatial variables in the late successional stand. These results suggest that environmental filtering likely influences the spatial distribution of AOA abundance in the early successional forest more than that in the late successional forest, whereas spatial dispersal may limit the distributions of AOA communities in the late successional subtropical forest. Our study sheds light on how to use spatial analyses to detect the responses of soil microbes to environmental changes and to evaluate the shifts in belowground microbial communities in conjunction with the trajectory of the aboveground forest recovery.

## Methods

### Site description and sampling

The study site is located in the Dinghushan Biosphere Reserve (DBR; 112°30′–112°33′ E, 23°09′–23°11′ N), Guangdong province, southeastern China. The reserve is characterized by a subtropical monsoon climate, with a mean annual temperature of 20.9 °C and annual precipitation of 1929 mm^37^. The soil is classified as lateritic red earth (or Oxisols based on the USDA soil taxonomy). The dominant vegetation type in the DBR is the subtropical evergreen broad-leaved forest (EBLF), which mainly consists of two stand types (Fig. 1): the primary EBLF stand with an age of about 400 years, and the secondary EBLF stand with an age of about 60 years.

In each stand, 31 quadrats (20 × 20 m^2^) were established for vegetation inventory and soil sampling. Three surface (0–10 cm) soil cores were collected using a soil auger (6.35 cm in diameter) around the center of each quadrat to form a composite sample (ca. 1.5 kg per sample). After being cleared of litter, animals and stones, these samples were sieved through a 2-mm mesh and divided into two parts. One part was used for soil chemical analysis; the other part was saved at −20 °C for DNA extraction and molecular analysis.

### Measurements of edaphic, vegetational and topographic properties

Soil pH testing was performed at a soil to water ratio of 1: 2.5 using a pH meter (Denver Instrument UB-7 pH/ev Meter, USA). Soil bulk density (BD) was determined by drying an intact soil column at 105 °C for 24 hours in a cylindrical stainless-steel container with known volume and weight. Soil organic matter (SOM), total nitrogen (TN), total phosphorus (TP) and total potassium (TK) were determined using the K_2_Cr_2_O_7_ oxidation, the semi-micro Kjeldahl, ascorbic acid colorimetric and atomic absorption methods, respectively, as described previously^38^. Available nitrogen (AN) was measured using the alkaline hydrolysis distillation method. Available phosphorus (AP) was extracted by NaHCO_3_ and measurement was performed on the filtrate by the molybdate-blue method. Available potassium (AK) was extracted with ammonium acetate and was measured by an atomic absorption spectrometer using ascorbic acid as a reductant.

The vegetation inventory was conducted by recording species name, diameter at breast height (DBH), height and coordinates of all free-standing trees. Vegetational attributes such as species diversity, species richness and tree density were presented by Shannon-Wiener index, species numbers and individual numbers in each quadrat, respectively (Table 2). The canopy tree species composition were presented by the sample scores along the first two principal component axes (i.e., veg-PC1 and veg-PC2) from a principal component analysis (PCA) on the tree community composition (tree height ≥10 m) (Supplementary Table S2, Table S3).

Four topographic properties were involved in our analysis, namely altitude, slope, convexity and aspect. The mean values of the four properties were calculated in each sampling plot by using the method described previously^39^. Aspect (degree from north) and convexity was calculated by using the method described as previous research^40^ and presented by dummy variables.

### DNA extraction and purification

Soil total DNA was extracted and purified using the method described previously^41^. In brief, 0.5 g of each soil sample was first washed with a buffer solution (135 mM NaCl, 2.7 mM KCl, 1.5 mM NaH_2_PO_4_, 8 mM Na_2_HPO_4_, 20 mM EDTA, pH = 7.4), and proteinase K (prK) was then applied to induce microbial cell lysis and the raw DNA then extracted. The raw DNA was purified by applying the following steps: i) incubated with potassium acetate (KAc) on ice and centrifuged at 4 °C, ii) precipitated overnight with polyethylene glycol 6000 (PEG) plus NaCl at 4 °C, and iii) washed three times with a mixed solution of phenol, chloroform and isoamyl alcohol (25:24:1, vol/vol/vol).

### AOA abundance quantification

Real-time PCR was conducted to determine AOA abundance on an ABI 7500 thermocycler system with the primer pair CrenamoA616r/CrenamoA23f^42^. Each reaction was performed in a 20 μl volume including 12.5 μl SYBR Premix Ex Taq (TaKaRa Biotechnology, Japan), 1 μl of each primer (10 mmol/L), and 2 μl of DNA template (1–10 ng). During the amplification, a three-step method was used with the following conditions: 95 °C for 30 s, 40 cycles of 5 s at 95 °C, 34 s at 53 °C, and 1 min at 72 °C. A standard curve was generated from a tenfold serial dilution (10^3^–10^8^ copies per μl) plasmids extracted from clones containing the archaeal *amoA* gene fragment. The number of gene copies was directly calculated from the concentration of extracted plasmid DNA, which was represented as AOA community abundance. The PCR efficiency and correlation coefficients (R^2^) for standard curves were 90.12% and 0.999, respectively.

### General statistical analysis

To compare the AOA abundance, soil physicochemical parameters, topographical features and vegetation characteristics between the two stands, an independent samples *t*-test was used if the assumptions of normality and homoscedasticity were satisfied. If these assumptions were not satisfied, a Non-parametric Mann-Whitney *U* test was employed. Significant level was set at *p* < 0.05. All these statistical tests were performed in R v. 3.1.0.

### Spatial analysis

To assess the degree of spatial dependence of the AOA abundance and its related environmental variables, empirical semivariograms were calculated using the following function:


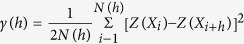


where γ (h) is the value of the semivariance for a lag distance h, N (h) is the number of data pairs divided by h, Z (x_i_) and Z (x_i + h_) represent the actual observed values of the variable x at the sites denoted by i and i + h, respectively. Since the spatial dependence decomposes at distances greater than half of the maximum distance between two samples^43^, we limited the calculation of the semivariograms for all of the variables to 250 m, and the omnidirectional semivariograms were calculated to generate sufficient observations within each lag distance class. Before calculating the semivariance, polynomial trend-surface regressions on each variable were performed using the method described as previous^44^. Residuals from the trend-surface regressions were used to generate the semivariograms and the confident envelops in the following spatial analyses.

To assess the significance of semivariance values in each lag class, a randomization procedure was used as described in previous research^45^. In each of the 10,000 randomizations, sample values were randomly selected and a semivariogram was calculated based on the selected data. A 95% confidence envelop was then obtained by calculating the 2.5 and 97.5 percentiles of the randomly generated semivariance values in each lag class. If the empirical semivariance lies within the 95% confidence envelope then it indicates that the tested variable is randomly distributed. However, if the semivariance lies below the lower edge or above the upper edge of the envelope, it suggests a significant positive or negative autocorrelation, respectively^46^. All semivariogram analyses were performed using the “geoR” package^47^ in R v. 3.1.0.

We also fitted the theoretical models (i.e., spherical, exponential, Gaussian and linear) to all the empirical semivariograms, and estimated the parameters (nugget, sill and range) by using the R package “gstat”^43^. The ordinary kriging process was performed using the Geostatistical Analyst Module extension in ArcGIS 10.2 (ESRI Inc., Redlands, CA, USA) using the semivariogram parameters obtained from the theoretical models fitted to the semivariograms. We examined the results of the semivariance and kriging analyses using normal transformed values, but they showed no significant differences with those obtained from the log-transformed values (for AOA abundance) and non-transformed values (for other variables). Therefore, we only present results for the latter.

### Variation partitioning

Multivariate variation-partitioning approach was conducted by using the R package “varpart” to quantify the relative contributions of edaphic, topographic, vegetation, and spatial attributes, and their potential intersections, to the spatial variation of AOA abundance. Spatial variables were obtained using the Moran’s eigenvector mapping (MEM) method^34^ with the “spacemakR” and “vegan” R packages^48^. MEM analysis revealed 10 and 11 spatial variables for the ES and LS stand, respectively (data not shown). The first through the third order terms for the topographical features (slope and altitude) were used to contrast the topographical matrix, and the first order term for the soil physicochemical and vegetation features to construct the edaphic and vegetational matrix. Before the variation partitioning analysis, the most significant influencial variables to AOA abundance in each group were chosen by permutational forward model selection by using the method developed previously^49^ with the R package “packfor”. In detail, a global test on each variable group was firstly performed, and then, the forward selection were carried out on the significant variable group with two stopping criteria: i) the alpha significance level and ii) the adjusted R^2^ of the reduced models did not exceed the adjusted R square of the global models. The variables that are strongly correlated were excluded during each forward selection model to avoid high multicollinearity. Statistical significances were assessed by 1000 permutation of the reduced models.

## Additional Information

**How to cite this article**: Chen, J. *et al.* Spatial distribution patterns of ammonia-oxidizing archaea abundance in subtropical forests at early and late successional stages. *Sci. Rep.* **5**, 16587; doi: 10.1038/srep16587 (2015).

## Supplementary Material

Supplementary Information

## Figures and Tables

**Table 1:** AOA abundance and soil properties in the early (ES) and late (LS) successional subtropical forest stands.

Properties	ES			LS		
	Mean	Range (min-max)	cv%	Mean	Range (min-max)	cv%
AOA abundance (Archaeal *amoA*	1.12^a^	0.005–8.33	168	1.77^a^	0.001–7.52	108
copy numbers×10^9^ g-1 dry soil)						
Soil bulk density (BD; g cm^−3^)	1.09^a^	0.92–1.20	7	0.99^b^	0.91–1.07	4
pH	3.80^a^	3.47–3.95	2	3.70^b^	3.58–3.80	2
Soil organic matter (SOM; g kg^−1^)	50.81^a^	42.61–66.31	11	73.76^b^	58.25–96.91	16
Total nitrogen (TN; g kg^−1^)	0.76^a^	0.47–1.22	30	1.82^b^	1.01–2.76	25
Total potassium (TK; g kg^−1^)	16.17^a^	8.45–21.98	17	20.70^b^	14.35–27.46	18
Total phosphorus (TP; g kg^−1^)	0.24^a^	0.21–0.31	11	0.34^b^	0.27–0.43	13
Available nitrogen (AN; mg kg^−1^)	174.14^a^	133.26–211.44	12	243.94^b^	206.32–280.92	9
Available potassium (AK; mg kg^−1^)	42.34^a^	33.75–54.72	14	72.32^b^	42.89–106.07	26
Available phosphorus (AP; mg kg^−1^)	2.63^a^	1.15–4.66	41	0.80^b^	0.49–1.17	29

Data are shown as mean, range and cv% (coefficient of variation) (n = 31). Means in the same row with different alphabetic superscripts (a, b) are significantly different between the two stands.

**Table 2:** Vegetation attributes and topographic features of the two evergreen broadleaved forest stands at early (ES) and late (LS) successional stages.

Attribute	ES			LS		
	Mean	Range (min-max)	cv%	Mean	Range (min-max)	cv%
Stand age (yr)	60	–	–	400	–	–
Altitude (m)	361.1^a^	278.2–463.2	15	342.5^a^	247.3–450.5	16
Mean slope (%)	29.1^a^	13.2–36.8	20	38.7^b^	16.3–57.9	18
Shannon-Wiener diversity	2.6^a^	1.6–3.1	14	2.7^a^	2.2–3.1	9
Species richness (# / quadrat)	25^a^	15–36	23	30^b^	15–43	22
Tree density (# / quadrat)	116^a^	45–204	36	142^b^	53–242	37
Tree height (m)	4.9^a^	1–30	12	4.6^b^	2–25	9
DBH (cm)	7.3^a^	1–54	27	5.8^b^	1–51	20

Data are shown as mean, range and cv % (coefficient of variation) (n = 31). Means in the same row with different alphabetic superscripts (a, b) are significantly different between the two stands.

## References

[b1] Bru D. *et al.* Determinants of the distribution of nitrogen-cycling microbial communities at the landscape scale. ISME J. 5, 532–542 (2011). 20703315 10.1038/ismej.2010.130 PMC3105713

[b2] Philippot L. *et al.* Mapping field-scale spatial patterns of size and activity of the denitrifier community. Environ. Microbiol. 11, 1518–1526 (2009). 19260937 10.1111/j.1462-2920.2009.01879.x

[b3] Drenovsky R. E., Steenwerth K. L., Jackson L. E. & Scow K. M. Land use and climatic factors structure regional patterns in soil microbial communities. Global Ecol. Biogeogr. 19, 27–39 (2010). 10.1111/j.1466-8238.2009.00486.x PMC3891896 24443643

[b4] Fraver S., White A. S. & Seymour R. S. Natural disturbance in an old-growth landscape of northern Maine, USA. J. Ecol. 97, 289–298 (2009).

[b5] Hofgaard A. *et al.* Role of disturbed vegetation in mapping the boreal zone in northern Eurasia. Appl. Veg. Sci. 13, 460–472 (2010).

[b6] Ettema C. H. & Wardle D. A. Spatial soil ecology. Trends Ecol. Evol. 17, 177–183 (2002).

[b7] Petra C. J., vanVliet Paul F. & Hendrix. Role of fauna in soil physical processes in Soil biological fertility - a key to sustainable land use in agriculture (eds Abbott, Lynette K., Murphy & Daniel V.) 37–59 (Springer, 2007).

[b8] Liang Y. *et al.* Community structure analysis of soil ammonia oxidizers during vegetation restoration in southwest China. J. Basic Microbiol. 54, 180–189 (2014). 23897748 10.1002/jobm.201300217

[b9] Banning N. C. *et al.* Soil microbial community successional patterns during forest ecosystem restoration. Appl. Environ. Microb. 77, 6158–6164 (2011). 10.1128/AEM.00764-11 PMC3165429 21724890

[b10] Zhang K. R., Dang H. S., Tan S. D., Wang Z. X. & Zhang Q. F. Vegetation community and soil characteristics of abandoned agricultural land and pine plantation in the Qinling Mountains, China. Forest Ecol. Manag. 259, 2036–2047 (2010).

[b11] Davidson E. A. *et al.* Nitrogen and phosphorus limitation of biomass growth in a tropical secondary forest. Ecol. Appl. 14, S150–S163 (2004).

[b12] Hedin L. O., Vitousek P. M. & Matson P. A. Nutrient losses over four million years of tropical forest development. Ecology 84, 2231–2255 (2003).

[b13] Lebrija-Trejos E., Perez-Garcia E. A., Meave J. A., Poorter L. & Bongers F. Environmental changes during secondary succession in a tropical dry forest in Mexico. J. Trop. Ecol. 27, 477–489 (2011).

[b14] Feng D., Zongsuo L., Xuexuan X., Xingchang Z. & Lun S. Spatial heterogeneity of soil nutrients and aboveground biomass in abandoned old-fields of Loess Hilly region in Northern Shaanxi, China. Acta. Ecologica. Sinica. 28, 13–22 (2008).

[b15] Hubbell S. P., Ahumada J. A., Condit R. & Foster R. B. Local neighborhood effects on long-term survival of individual trees in a neotropical forest. Ecol. Res. 16, (2001).

[b16] Rantalainen M. L., Fritze H., Haimi J., Pennanen T. & Setala H. Colonisation of newly established habitats by soil decomposer organisms: the effect of habitat corridors in relation to colonisation distance and habitat size. Appl. Soil Ecol. 28, 67–77 (2005).

[b17] Cho J. C. & Tiedje J. M. Biogeography and degree of endemicity of fluorescent *Pseudomonas* strains in soil. Appl. Environ. Microb. 66, 5448–5456 (2000). 10.1128/aem.66.12.5448-5456.2000 PMC92480 11097926

[b18] Ramette A. & Tiedje J. M. Multiscale responses of microbial life to spatial distance and environmental heterogeneity in a patchy ecosystem. P. Natl. Acad. Sci. USA 104, 2761–2766 (2007). 10.1073/pnas.0610671104 PMC1815255 17296935

[b19] Fierer N. & Ladau J. Predicting microbial distributions in space and time. Nat. Methods 9, 549–550 (2012). 22669651 10.1038/nmeth.2041

[b20] Wankel S. D., Mosier A. C., Hansel C. M., Paytan A. & Francis C. A. Spatial variability in nitrification rates and ammonia-oxidizing microbial communities in the agriculturally impacted Elkhorn Slough Estuary, California. Appl. Environ. Microb. 77, 269–280 (2011). 10.1128/AEM.01318-10 PMC3019697 21057023

[b21] Gubry-Rangin C. *et al.* Niche specialization of terrestrial archaeal ammonia oxidizers. P. Natl. Acad. Sci. USA 108, 21206–21211 (2011). 10.1073/pnas.1109000108 PMC3248517 22158986

[b22] Isobe K. *et al.* High abundance of ammonia-oxidizing archaea in acidified subtropical forest soils in southern China after long-term N deposition. FEMS Microbiol. Ecol. 80, 193–203 (2012). 22224831 10.1111/j.1574-6941.2011.01294.x

[b23] Erguder T. H., Boon N., Wittebolle L., Marzorati M. & Verstraete W. Environmental factors shaping the ecological niches of ammonia-oxidizing archaea. FEMS Microbiol. Rev. 33, 855–869 (2009). 19453522 10.1111/j.1574-6976.2009.00179.x

[b24] Kester R. A., deBoer W. & Laanbroek H. J. Production of NO and N_2_O by pure cultures of nitrifying and denitrifying bacteria during changes in aeration. Appl. Environ. Microb. 63, 3872–3877 (1997). 10.1128/aem.63.10.3872-3877.1997 PMC1389263 16535707

[b25] Wessen E. *et al.* Spatial distribution of ammonia-oxidizing bacteria and archaea across a 44-hectare farm related to ecosystem functioning. ISME J. 5, 1213–1225 (2011). 21228891 10.1038/ismej.2010.206 PMC3146283

[b26] Bates S. T. *et al.* Examining the global distribution of dominant archaeal populations in soil. ISME J. 5, 908–917 (2011). 21085198 10.1038/ismej.2010.171 PMC3105767

[b27] Wang J. T. *et al.* Altitudinal distribution patterns of soil bacterial and archaeal communities along Mt. Shegyla on the Tibetan Plateau. Microb. Ecol. 69, 135–145 (2015). 25074792 10.1007/s00248-014-0465-7

[b28] Angel R., Soares M. I. M., Ungar E. D. & Gillor O. Biogeography of soil archaea and bacterial along a steep precipitation gradient. ISME J. 4, 553–563 (2011). 20033070 10.1038/ismej.2009.136

[b29] Walsh D. A., Papke R. T. & Doolittle W. F. Archaeal diversity along a soil salinity gradient prone to disturbance. Environ. Microbiol. 7, 1655–1666 (2005). 16156738 10.1111/j.1462-2920.2005.00864.x

[b30] Li Y. L. *et al.* Changes in forest soil properties in different successional stages in lower tropical China. PLoS One 8, (2013). 10.1371/journal.pone.0081359 PMC3828269 24244738

[b31] Merila P. *et al.* Soil organic matter quality as a link between microbial community structure and vegetation composition along a successional gradient in a boreal forest. Appl. Soil Ecol. 46, 259–267 (2010).

[b32] Stopnisek N. *et al.* Thaumarchaeal ammonia oxidation in an acidic forest peat soil is not influenced by ammonium amendment. Appl. Environ. Microb. 76, 7626–7634 (2010). 10.1128/AEM.00595-10 PMC2976176 20889787

[b33] Zhalnina K., de Quadros P. D., Camargo F. A. & Triplett E. W. Drivers of archaeal ammonia-oxidizing communities in soil. Front. Microbiol. 3, 210 (2012). 22715335 10.3389/fmicb.2012.00210 PMC3375578

[b34] Gilbert B. & Bennett J. R. Partitioning variation in ecological communities: do the numbers add up? J. Appl. Ecol. 47, 1071–1082 (2010).

[b35] Smith T. W. & Lundholm J. T. Variation partitioning as a tool to distinguish between niche and neutral processes. Ecography 33, 648–655 (2010).

[b36] Diniz J. A. F. *et al.* Spatial autocorrelation analysis allows disentangling the balance between neutral and niche processes in metacommunities. Oikos 121, 201–210 (2012).

[b37] Wang Z. F. *et al.* Genetic groups in the common plant species *Castanopsis chinensis* and their associations with topographic habitats. Oikos 121, 2044–2051 (2012).

[b38] Yu D. F. *et al.* In Soil physical and chemical analysis and description of soil profiles (eds. Liu G. S., Jiang N. H., Zhang L. D. & liu Z. L.) 24–40 (China Standards Press, Beijing, 1996).

[b39] Harms K. E., Condit R., Hubbell S. P. & Foster R. B. Habitat associations of trees and shrubs in a 50-ha neotropical forest plot. J. Ecol. 89, 947–959 (2001).

[b40] Lin G. J. *et al.* Separating the effects of environment and space on tree species distribution: from population to community. PLoS One 8, e56171 (2013). 23409151 10.1371/journal.pone.0056171 PMC3568135

[b41] Krsek M. & Wellington E. M. H. Comparison of different methods for the isolation and purification of total community DNA from soil. J. Microbiol. Meth. 39, 1–16 (1999). 10.1016/s0167-7012(99)00093-7 10579502

[b42] Zhang L. M., Hu H. W., Shen J. P. & He J. Z. Ammonia-oxidizing archaea have more important role than ammonia-oxidizing bacteria in ammonia oxidation of strongly acidic soils. ISME J. 6, 1032–1045 (2012). 22134644 10.1038/ismej.2011.168 PMC3329103

[b43] Cressie N. A. C. Geostatistics In Statistics for spatial data (J.Wiley, 1993).

[b44] John R. *et al.* Soil nutrients influence spatial distributions of tropical tree species. P. Natl. Acad. Sci. USA 104, 864–869 (2007). 10.1073/pnas.0604666104 PMC1783405 17215353

[b45] Diggle P. J. & Ribeiro P. J. Classical parameter estimation In Model-based geostatistics (Springer, 2007).

[b46] Siefert A. Spatial patterns of functional divergence in old-field plant communities. Oikos 121, 907–914 (2012).

[b47] Ribeiro P. & Diggle P. geo{R}: a package for geostatistical analysis. R-NEWS 1, 14–18 (2001).

[b48] Dray S., Legendre P. & Peres-Neto P. R. Spatial modelling: a comprehensive framework for principal coordinate analysis of neighbour matrices (PCNM). Ecol. Model. 196, 483–493 (2006).

[b49] Blanchet F. G., Legendre P. & Borcard D. Forward selection of explanatory variables. Ecology 89, 2623–2632 (2008). 18831183 10.1890/07-0986.1

