# Supplementary material for: Spatial distribution patterns of ammonia-oxidizing archaea abundance in subtropical forests at early and late successional stages
Source: Sci Rep. 2015 Nov 13;5:16587. doi: 10.1038/srep16587 (PMC4643239; doi:10.1038/srep16587)
Supplement: Supplementary Information [file srep16587-s1.doc]

**Spatial distribution patterns of ammonia-oxidizing archaea abundance in subtropical forests at early and late successional stages**

**Jie Chen ^1,2^, Hui Zhang ^1^, Wei Liu ^1^, Juyu Lian ^1^, Wanhui Ye** **^1^, Weijun Shen^1*^**

**^1^ Key Laboratory of Vegetation Restoration and Management of Degraded Ecosystems, South China Botanical Garden, Chinese Academy of Sciences,**

723 Xinke Rd. Tianhe District, Guangzhou 510650, China

**^2^** University of Chinese Academy of Sciences, Beijing 100049, China

^*^Corresponding author: Dr. Weijun Shen

Key Laboratory of Vegetation Restoration and Management of Degraded Ecosystems,

South China Botanical Garden, Chinese Academy of Sciences

723 Xingke Road, Tianhe District

Guangzhou 510650,

China

Tel.: + 86 20 3725 2950;

Fax: + 86 20 3725 2950;

Email: [shenweij@scbg.ac.cn](mailto:shenweij@scbg.ac.cn)

**Supplementary Table S1** Semivariogram parameter estimates of Gau (Gaussian) and Shp (Spherical) variogram models) for environmental variables and log-transformed AOA abundance in the ES and LS stand.

| Variable | ES | | | | | |  | LS | | | | | | |
| --- | --- | --- | --- | --- | --- | --- | --- | --- | --- | --- | --- | --- | --- | --- |
|  | Trend  order | Model | Nugget | Partial  sill | Range | Normalized  sill (%) |  | Trend  order | Model | | Nugget | Partial  sill | Range | Normalized  sill (%) |
| LogAOA | NS | Sph | 0.167 | 1.20 | 65.9 | 87.8 |  | NS | Gau | 0.38 | | 1.38 | 90.6 | 78.4 |
| BD | 2 | Gau | 1.00E-06 | 2.00E-03 | 69.2 | 99.9 |  | 2 | Sph | 1.00E-06 | | 8.80E-04 | 76.9 | 99.9 |
| pH | 1 | Gau | 5.57E-05 | 9.80E-04 | 68.9 | 94.6 |  | 2 | Gau | 2.80E-04 | | 1.80E-03 | 89.3 | 86.5 |
| SOM | NS | Gau | 1.70E-03 | 0.18 | 125.9 | 99.0 |  | 2 | Sph | 2.90E-02 | | 0.11 | 150.3 | 79.3 |
| TN | 1 | Gau | 1.00E-06 | 6.61E-05 | 54.8 | 98.5 |  | 2 | Sph | 1.00E-06 | | 4.90E-02 | 90.2 | 100.0 |
| TK | NS | Gau | 1.00E-06 | 10.41 | 88.4 | 99.9 |  | 2 | Gau | 0.85 | | 5.61 | 104.4 | 86.8 |
| TP | 2 | Gau | 1.34E-05 | 4.27E-04 | 148.6 | 97.0 |  | 2 | NS | NS | | NS | NS | NS |
| AN | 2 | Gau | 23.56 | 135.56 | 155.3 | 85.2 |  | 2 | NS | NS | | NS | NS | NS |
| AK | 2 | Gau | 6.10E-04 | 1.50E-03 | 82.5 | 71.1 |  | 2 | Sph | 31.39 | | 88.30 | 57.6 | 73.7 |
| AP | 1 | Gau | 2.96E-04 | 0.05 | 203.6 | 99.4 |  | 2 | Gau | 5.93E-06 | | 3.88E-03 | 66.2 | 99.8 |
| Altitude | 2 | Gau | 10.85 | 78.20 | 74.0 | 87.8 |  | 2 | Sph | 5.71 | | 89.35 | 61.1 | 94.0 |
| Slope | NA | Gau | 6.25 | 36.16 | 112.5 | 85.3 |  | 2 | NS | NS | | NS | NS | NS |
| Species diversity | NS | NS | NS | NS | NS | NS |  | NS | NS | NS | | NS | NS | NS |
| Species richness | NS | NS | NS | NS | NS | NS |  | 2 | Sph | 13.19 | | 15.79 | 52.5 | 54.5 |
| Tree density | 1 | NS | NS | NS | NS | NS |  | 2 | Sph | 800.73 | | 1586.50 | 51.8 | 66.5 |
| Tree height | NS | NS | NS | NS | NS | NS |  | NS | Sph | 3.80E-02 | | 0.174 | 64.0 | 82.1 |
| DBH | NS | NS | NS | NS | NS | NS |  | NS | Sph | 0.45 | | 1.01 | 76.0 | 69.1 |
| Veg-PC1 | NS | Gau | 0.52 | 1.62 | 74.8 | 75.7 |  | NS | NS | NS | | NS | NS | NS |
| Veg-PC2 | NS | NS | NS | NS | NS | NS |  | 1 | NS | NS | | NS | NS | NS |

Abbreviations: AOA, Archaeal *amoA* gene copy numbers per gram of dry soil; BD, soil bulk density; SOM, soil total organic matter; TN, soil total nitrogen; TK, soil total potassium; TP, soil total phosphorus; AN, soil available nitrogen; AK, soil available potassium; AP, soil available phosphorus; Species diversity: Shannon-Wiener diversity; veg-PC1, veg-PC2, the first two principle components of the canopy layer.

Residuals from the trend surface regressions were used to calculate the empirical vairograms. Trend order is the order of the polynomial regression, and “NA” means no significant trends and fitted semivariogram models were detected.

**Supplementary Table S2** Species loadings along the first and second principal component of the vegetation composition belong to the canopy layer in the early successional (ES) and late successional (LS) stand.

| ES | | |  | LS | | |
| --- | --- | --- | --- | --- | --- | --- |
| Species | PC1 score | PC2 score |  | Species | PC1 score | PC2 score |
| *Machilus chinensis* | 0.072939 | -0.4009 |  | *Gironniera subaequalis* | 0.576034 | 0.108536 |
| *Craibiodendron kwangtungense* | 0.349383 | -0.4956 |  | *Machilus chinensis* | 0.608527 | -0.32049 |
| *Pinus massoniana* | 0.521429 | 0.318624 |  | *Engelhardtia roxburghiana* | 0.166824 | -0.64455 |
| *Engelhardtia roxburghiana* | -0.40072 | 0.446307 |  | *Acmena acuminatissima* | -0.31268 | 0.083215 |
| *Schima superba* | 0.521195 | -0.01345 |  | *Schima superba* | -0.40078 | -0.55946 |
| *Castanopsis chinensis* | -0.4105 | -0.54108 |  | *Castanopsis chinensis* | 0.107958 | 0.387481 |
| Proportion of Variance (*%*) | 33.14 | 21.68 |  | Proportion of Variance (*%*) | 24.98 | 23.54 |
| Cumulative Proportion (*%*) | 33.14 | 54.83 |  | Cumulative Proportion (*%*) | 24.98 | 48.52 |

**Supplementary Table S3** The first two principal components of the canopy layer composition in the early successional (ES) and late successional (LS) stand.

| Sampling quadrat code | ES | |  | LS | |
| --- | --- | --- | --- | --- | --- |
|  | PC1 | PC2 |  | PC1 | PC2 |
| 1 | 0.309761 | 0.211598 |  | 0.095156 | 1.593267 |
| 2 | -0.56961 | 0.337583 |  | -0.16585 | 0.656478 |
| 3 | -1.17839 | -0.83379 |  | -0.5034 | 0.746312 |
| 4 | -1.56293 | -1.34066 |  | 0.182156 | 1.90553 |
| 5 | 0.021284 | 0.200285 |  | -1.03225 | -0.55296 |
| 6 | -0.20542 | -3.27034 |  | -0.07885 | 0.968741 |
| 7 | -0.16785 | -1.21152 |  | -0.25285 | 0.344215 |
| 8 | -0.42102 | 0.111266 |  | -0.07885 | 0.968741 |
| 9 | -1.4298 | 0.963955 |  | -0.16585 | 0.656478 |
| 10 | -0.19987 | 0.155775 |  | -0.5904 | 0.434049 |
| 11 | 0.877989 | -0.25515 |  | 2.136824 | -0.0516 |
| 12 | 0.887653 | 0.813909 |  | -0.68605 | -0.2605 |
| 13 | -0.69901 | 1.064287 |  | -1.26552 | 0.613718 |
| 14 | 0.98803 | 1.033799 |  | -1.69872 | 0.009 |
| 15 | 0.446624 | -0.08339 |  | 3.092751 | 0.577828 |
| 16 | 2.366513 | -1.78276 |  | 2.136824 | -0.0516 |
| 17 | 3.085109 | -0.2188 |  | 0.497139 | -0.88364 |
| 18 | 0.883339 | 1.08957 |  | -0.5904 | 0.434049 |
| 19 | -1.33251 | 0.563847 |  | -0.16585 | 0.656478 |
| 20 | -1.55489 | 1.414998 |  | -1.02361 | -0.17067 |
| 21 | 3.616177 | 1.062131 |  | 0.953636 | 0.57154 |
| 22 | 0.943163 | -2.19104 |  | -1.69872 | 0.009 |
| 23 | -0.67121 | 1.013352 |  | -0.93851 | -3.84201 |
| 24 | -0.16035 | 0.173515 |  | -1.11925 | -0.86522 |
| 25 | 1.448478 | 0.002824 |  | 0.853786 | 0.747703 |
| 26 | 0.768156 | 0.181007 |  | -0.27362 | -1.39852 |
| 27 | -2.32583 | -1.11439 |  | -0.16585 | 0.656478 |
| 28 | -1.81125 | 1.077087 |  | -1.46545 | -1.15768 |
| 29 | -1.81685 | -0.65451 |  | 2.81447 | -2.96186 |
| 30 | 0.228216 | 1.880062 |  | 0.953636 | 0.57154 |
| 31 | -0.76371 | -0.3945 |  | 0.243447 | -0.9249 |

**
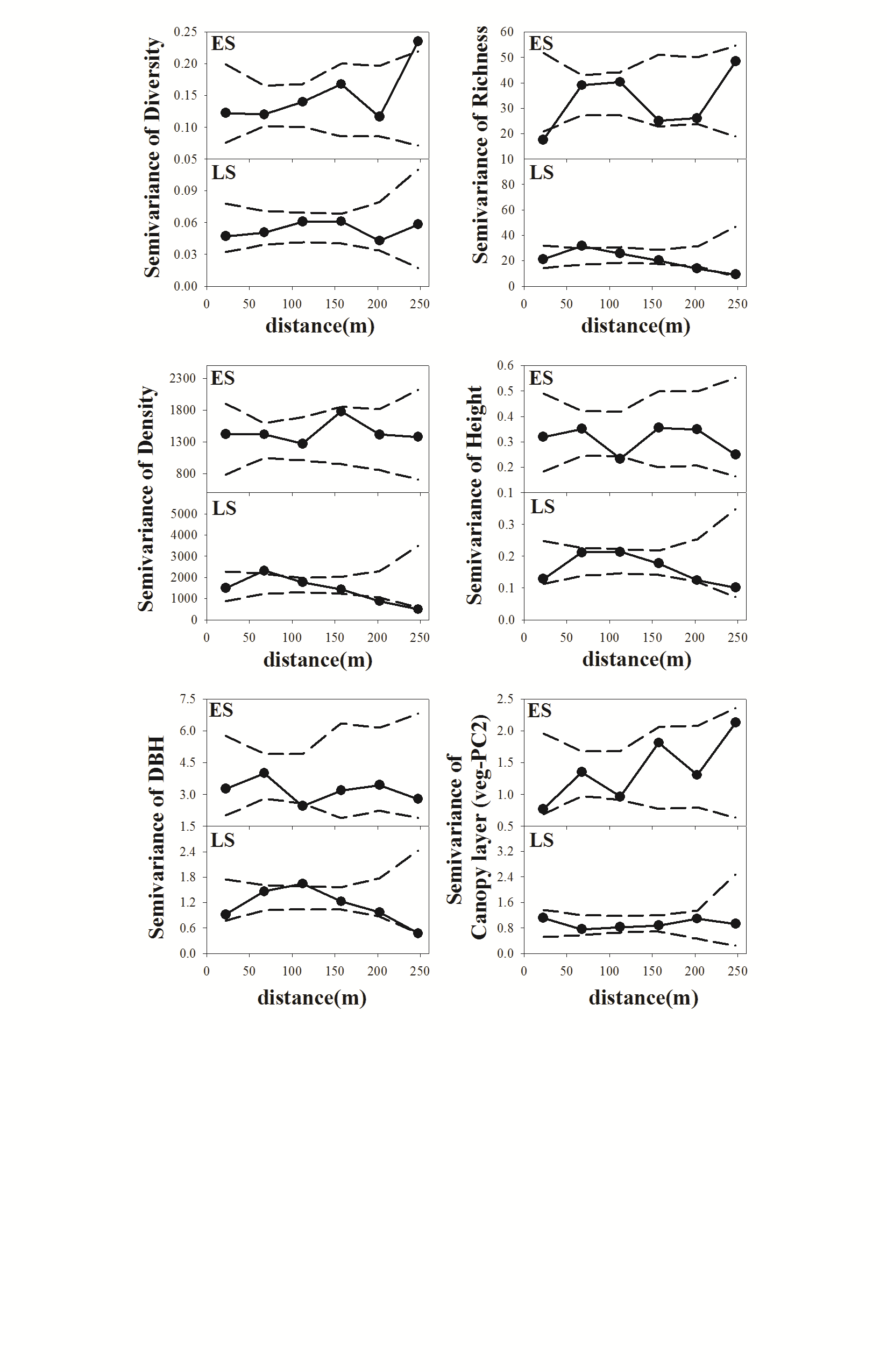
**

**
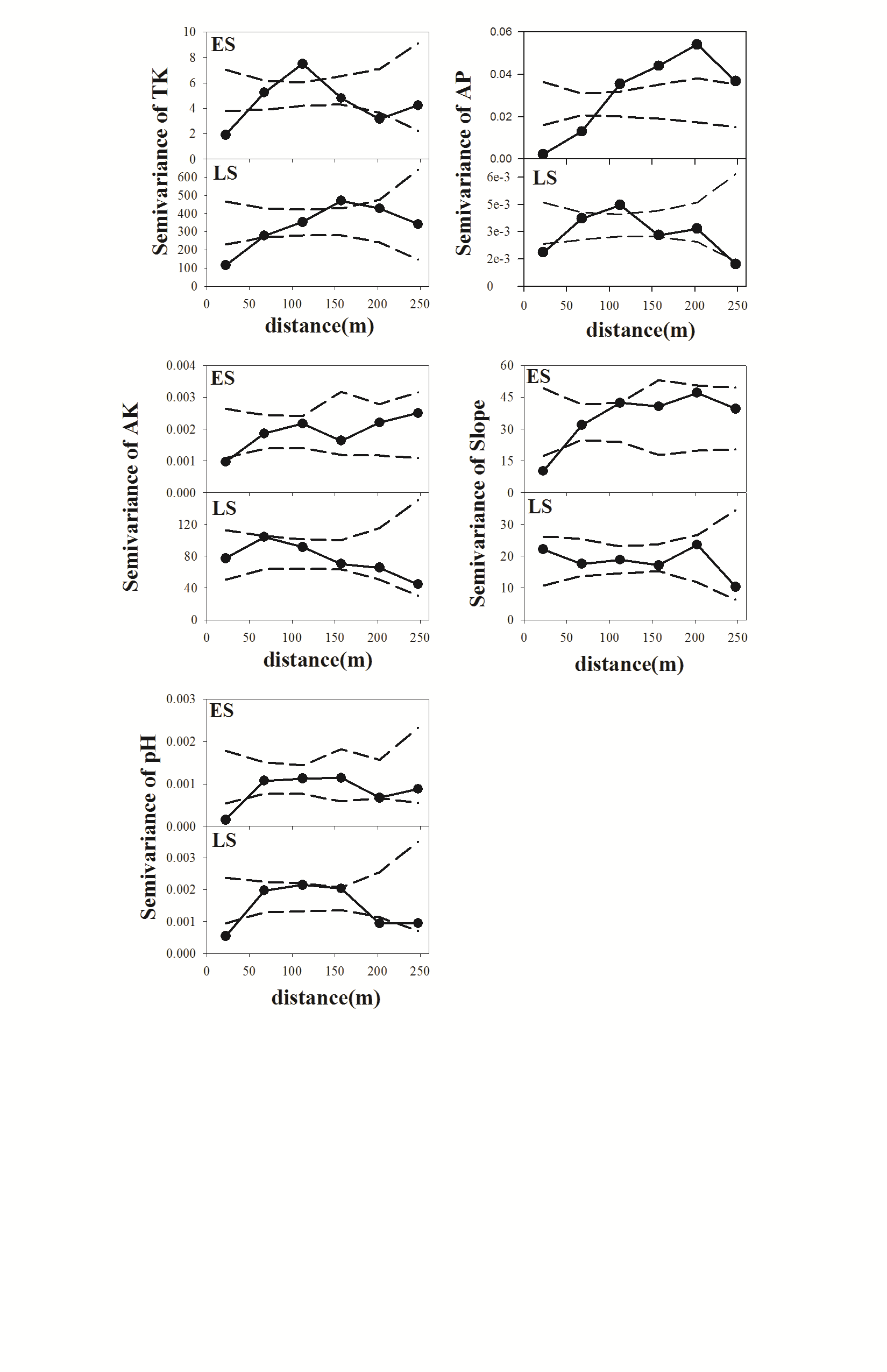
**

**Supplementary Figure S1** Spatial distribution pattern of environmental variables in

the ES and LS stands. Observed semivariance was represented by solid circles, and dashed lines define 95% confidence envelope based on 10,000 randomizations of the data. Semivariance values below confidence envelope indicate positive spatial autocorrelation (less divergence than expected at random); values above confidence envelope indicate negative spatial autocorrelation (more divergence than expected at random).

**
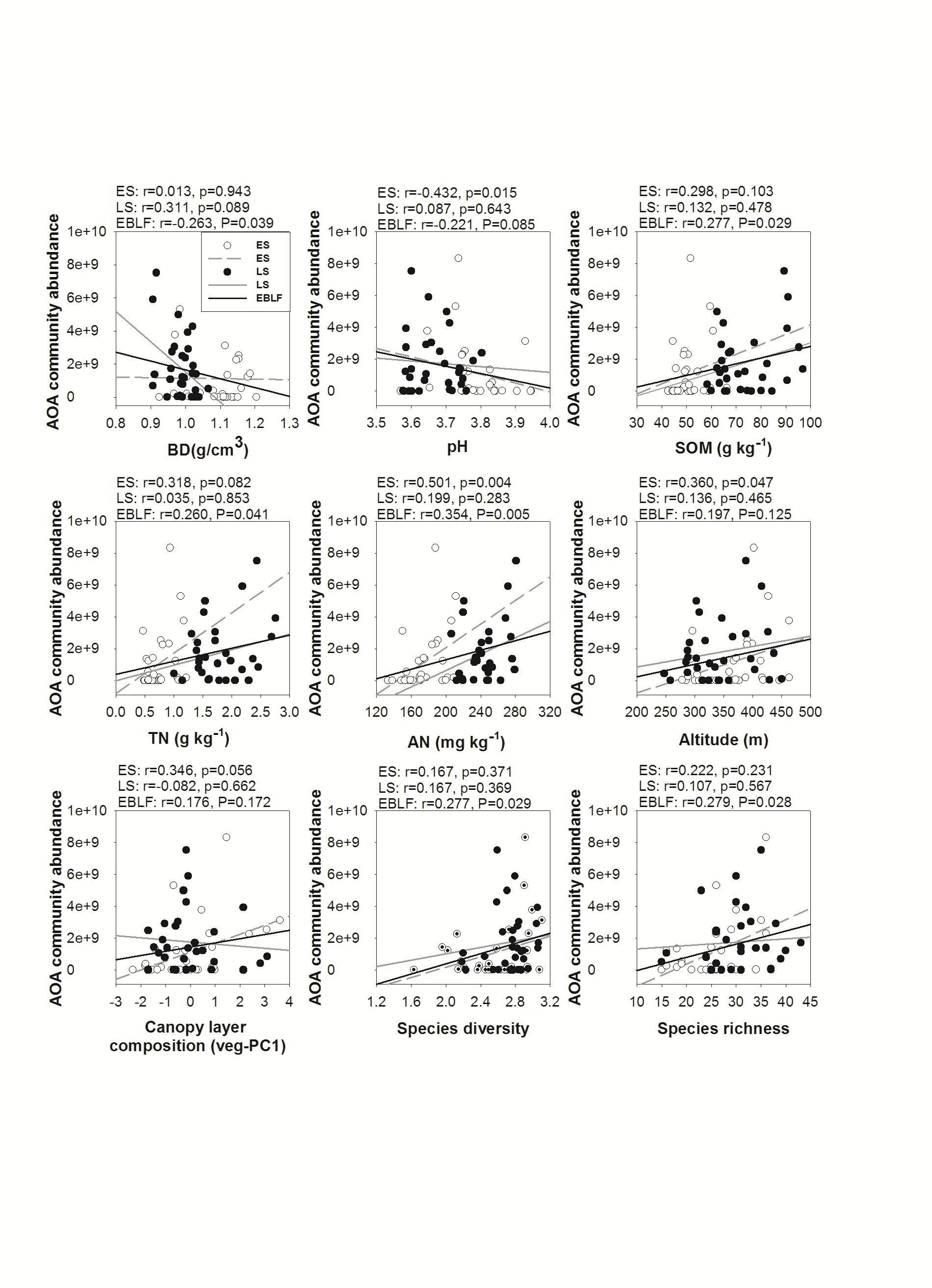
**

**Supplementary Figure S2** Graphs illustrating the relationship between AOA abundance and environmental variables for the ES stand, LS stand and the EBLF. r and *P* represent the spearman correlation coefficient and its associated significance level (*P*-0.05).
